# Differential proteome profiling of bacterial culture supernatants reveals candidates for the induction of oral immune priming in the red flour beetle

**DOI:** 10.1098/rsbl.2023.0322

**Published:** 2023-11-01

**Authors:** Zoe Marie Länger, Moritz Baur, Ana Korša, Jürgen Eirich, Ana Sofia Lindeza, Caroline Zanchi, Iris Finkemeier, Joachim Kurtz

**Affiliations:** ^1^ Institute for Evolution and Biodiversity, University of Münster, Hüfferstraße 1, 48149 Münster, Germany; ^2^ Institute of Plant Biology and Biotechnology, University of Münster, Schlossplatz 7, 48149 Münster, Germany

**Keywords:** insects, immune priming, *Tribolium castaneum*, *Bacillus thuringiensis*, Cry3aa, proteome

## Abstract

Most organisms are host to symbionts and pathogens, which led to the evolution of immune strategies to prevent harm. Whilst the immune defences of vertebrates are classically divided into innate and adaptive, insects lack specialized cells involved in adaptive immunity, but have been shown to exhibit immune priming: the enhanced survival upon infection after a first exposure to the same pathogen or pathogen-derived components. An important piece of the puzzle are the pathogen-associated molecules that induce these immune priming responses. Here, we make use of the model system consisting of the red flour beetle (*Tribolium castaneum*) and its bacterial pathogen *Bacillus thuringiensis*, to compare the proteomes of culture supernatants of two closely related *B. thuringiensis* strains that either induce priming via the oral route, or not. Among the proteins that might be immunostimulatory to *T. castaneum,* we identify the Cry3Aa toxin, an important plasmid-encoded virulence factor of *B. thuringiensis*. In further priming–infection assays we test the relevance of Cry-carrying plasmids for immune priming. Our findings provide valuable insights for future studies to perform experiments on the mechanisms and evolution of immune priming.

## 1. Introduction

In insects the degree of specificity of immune responses varies substantially, from non-specific to a high degree of specificity, even regarding closely related pathogen strains [1–3]. There is evidence for forms of immune memory called ‘immune priming’, which result in enhanced survival upon infection with a pathogen, after a preliminary encounter with that pathogen or pathogen-derived cues. Immune priming could provide similar benefits for the insect host as the adaptive response does for the vertebrate host, if increased protection is specific, long-lasting, and, as mentioned in some postulations, biphasic [4–7]. Cases of immune priming have been described in many insect hosts, but in most cases the exact mechanisms of these phenomena are not yet fully understood [8–12].

The red flour beetle *Tribolium castaneum* and its pathogen *Bacillus thuringiensis* bv. *tenebrionis* (*Btt*) provide a well-established model system to study immune priming. Feeding *T. castaneum* larvae with a mix of flour and filter-sterilized supernatant of sporulated *Btt* results in increased survival upon infection with *Btt* spores and toxin mix [13]. This oral priming might be specific and involves the differential expression of genes targeting orally ingested pathogens [14,15]. Furthermore, the gut microbiota was shown to be affected by such a treatment and essential for oral priming [16,17].

Here, we conducted mass-spectrometry-based proteomic analyses to compare the supernatant composition of *Btt* cultures, which induce immune priming, with those of a non-pathogenic *B. thuringiensis* strain, *Bt407-*, which do not induce immune priming. We identified the Cry3Aa toxin as a candidate elicitor and further characterized its potential involvement in oral immune priming, using priming–infection experiments. To our knowledge, this is the first study that describes proteins that may elicit oral immune priming.

## 2. Material and methods

### (a) Model organisms

We used *T. castaneum* from the outbred population ‘Croatia 1’ (CRO 1) [18]. For rearing conditions see electronic supplementary material. *Bacillus thuringiensis* bv *tenebrionis* (*Btt*) was purchased from the BGSC (Ohio State University, USA). *B. thuringiensis 407 Cry-* (hereafter *Bt407-*), was kindly provided by Dr Christina Nielsen-Leroux, INRAE (France), see [18]. *B. thuringiensis 407gfp-neocry +* (hereafter *Bt407+*) obtained the Cry3Aa plasmid via conjugation [18]. *B. thuringiensis Cry-* (hereafter *Btt-*) was cured of the Cry3Aa encoding plasmid by serial-passaging (electronic supplementary material, table S1, and figures S1 and S2).

### (b) Preparation of priming and infection diets

We prepared spore cultures as described in [18] with some minor modifications (electronic supplementary material). For priming diets, we centrifuged grown cultures twice (2604 rcf, 15 min). We filtered the supernatants through 0.45 µm and 0.22 µm filters to remove bacterial cells and mixed the filtered supernatants (or fresh liquid medium for control diets) with 0.15 g flour ml^−1^. Then we pipetted 10 µl into each well of 96-well flat bottom plates and dried them at 30°C overnight. For studying how culture age influences the ability of *Btt* supernatants to induce priming, we centrifuged cultures after 12 h and continuously on days 1–7, respectively, and prepared priming diets as described above. For infection diets, we centrifuged grown *Btt* cultures after 7 days (2604 rcf, 15 min), washed the spore pellet in 15 ml PBS, centrifuged and fetched the pellet in 5 ml PBS. We adjusted the concentration to 1 × 10^10^ spores ml^−1^ in PBS and mixed 0.15 g flour ml^−1^ of the spore–PBS mix. We pipetted 10 µl into each well of 96-well flat bottom plates and dried them at 30°C overnight.

### (c) Priming and infection experiments

We performed priming and infection of *T. castaneum* larvae as described in [18]. Shortly, we individualized larvae on day 15 after oviposition onto priming diets or control diets. After 24 h we transferred the larvae onto PBS diets (0.15 g flour ml^−1^ PBS) and left them for 4 days at standard conditions. Finally, we transferred the larvae onto infection or PBS diets and recorded survival for 5 days. We tested all treatment levels simultaneously on each 96-well plate in an experiment and repeated the experiments in blocks (for sample size and number of blocks see electronic electronic supplementary material, file S1, supplementary method).

### (d) Proteomics

To identify differentially enriched proteins in the supernatants of *Btt* and *Bt407-* cultures, we performed high resolution LC-MS/MS analysis by using an EASY-nLC 1200 (Thermo Fisher) coupled to an Exploris 480 mass spectrometer (Thermo Fisher). We separated peptides on 20 cm frit-less silica emitters (CoAnn Technologies, 0.75 µm inner diameter), packed in-house with reversed-phase ReproSil-Pur C_18_ AQ 1.9 µm resin (Dr Maisch) and kept the column constantly at 50°C. We acquired the mass spectra in data-dependent acquisition mode as outlined in Sindlinger *et al*. [19]. We processed the raw data, by using the MaxQuant software version 2.0.3.0 [20]. MS/MS spectra were assigned to a custom *Btt* proteome assembly (Dr Heiko Liesegang, Institute of Microbiology and Genetics, University of Göttingen, unpublished) with default settings and match between runs; LFQ and iBAQ options were enabled. For further downstream analysis we used R [21]. After log_2_ transformation, we imputed missing LFQ intensities based on quantile regression using imputeLCMD. To test for differential expression, we used LIMMA [22,23]. Raw data were uploaded to the JPOST repository [24].

### (e) Statistical analysis

We used R [21] and RStudio [25] for statistical analyses. For survival analysis, we used Cox proportional hazards model with one random factor using the ‘coxme’ function from the ‘coxme’ package [26,27]. The models therefore tested survival of the larvae according to the priming treatment as an explanatory variable, as well as either the experimental batch or the 96-well plate as a random factor [28,29]. To compare the priming treatment levels, we set the control treatment (*Bt* medium) as the intercept, and retrieved the estimates and 95% confidence intervals (95% CI) from contrasts in the model summary. We plotted the hazard ratios (HR) of the treatment levels versus the control, with the 95% CI around the HR with ‘ggplot2’ [30]. Final editing of the figures was done in Inkscape [31].

## 3. Results and discussion

**Figure 1.**
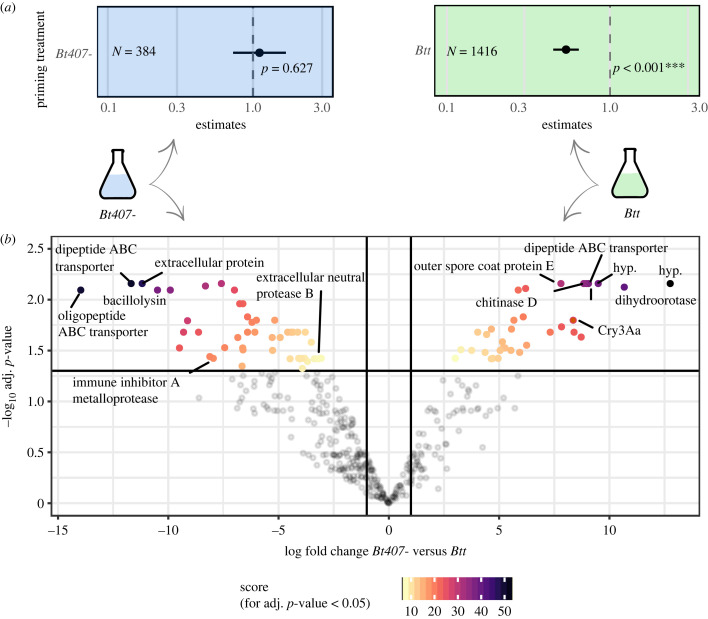
Proteomic profiles of two supernatants differing in their ability to induce immune priming. (*a*) Hazard ratios (HR) of *T. castaneum* larvae upon exposure to *Btt* spores after priming with *Bt407-* or *Btt* filter-sterilized spore culture supernatants (*Bt407-*: *χ*^2^ = 0.6229, d.f = 1, *p* = 0.627; *Btt*: *χ*^2^ = 41.62, d.f. = 1, *p* ≤ 0.001) compared to the control treatment (fresh *Bt* medium). Bars represent the 95% confidence intervals (95% CI). HR < 1 and no overlap of 95% CI with 1 indicate increased survival of the priming treatment. (*b*) Volcano plot for analysis of differential protein log_2_ fold change in LFQ intensities were plotted versus Benjamini–Hochberg adjusted *p*-values of LIMMA. Log_2_ fold change of ± 1 is indicated as vertical solid lines and an adjusted *p*-value of 0.05 as horizontal line. For protein groups with a log_2_ fold change > 1 or < −1 and an adjusted *p*-value < 0.05 the product of these values is shown as an enrichment score and used to colour the respective items. hyp.: hypothetical protein. Labelled proteins: highly differentially expressed or mentioned in the text.

We compared the proteomes of the supernatants of two *B. thuringiensis* strains, which were previously shown to differ in their ability to induce oral immune priming in *T. castaneum* larvae. We confirmed the described priming phenotype: exposure to supernatants of *Btt* led to an increased survival of *T. castaneum* larvae upon secondary infection with *Btt* spores, whereas exposure to supernatants of *Bt407-* did not (compared to medium control, figure 1*a*, electronic supplementary material, figures S3 and S4). Differential proteome profiling identified protein groups that were either uniquely expressed in one, or highly differentially expressed between *Btt* or *Bt407*- supernatants, respectively (*Btt*: 34 protein groups, *Bt407-*: 46 protein groups, figure 1*b*, electronic supplementary material, tables S2 and S3).

Among the most differentially expressed proteins in the *Btt* supernatant was the known coleopteran virulence factor Cry3Aa. Different strains of *B. thuringiensis* express different Cry toxins, which are specifically harmful to their respective host [32]. Therefore, it might be an evolutionary advantage for the insect immune system to specifically recognize and counteract them [33].

**Figure 2.**
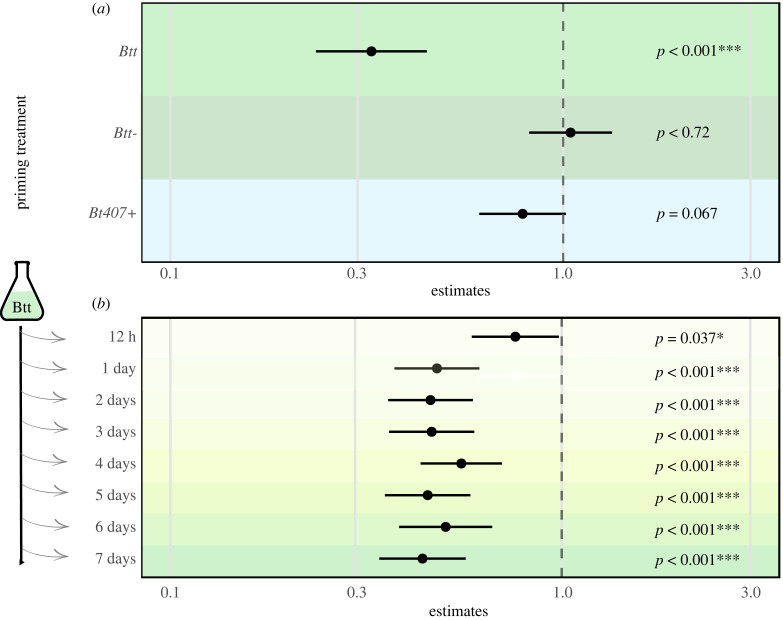
Hazard ratios (HR) of *T. castaneum* larvae from the different levels of the priming treatment versus the control treatment (fresh *Bt* medium). Bars represent the 95% confidence intervals (95% CI) around the HR. HR < 1 and no overlap of 95% CI with 1 indicate an increased survival of the priming treatment. (*a*) HR in *T. castaneum* larvae upon exposure to *Btt* spores after priming with filter-sterilized spore culture supernatants derived from *Btt*, *Btt-* (*Btt* strain cured of the Cry3Aa carrying plasmid) or *Bt407+* (*Bt407* strain with the Cry3Aa encoding plasmid). *Btt* supernatant served as the positive control for priming. There was an overall significant effect of the priming treatment on survival (*χ*^2^ = 70.6, d.f. = 3, *p* ≤ 0.001) with *Btt* having a significant effect and *Bt407 +* showing a trend to induce a better survival than priming with *Btt*-. (*b*) HR caused by *Btt* filter-sterilized spore culture supernatant harvested at different time points during the culture of *Btt* versus *Bt medium*. Priming with *Btt* supernatant from each time point had a significant effect on survival to *Btt* (*χ*^2^ = 75.6, d.f. = 8, *p* ≤ 0.001).

To investigate the potential role of Cry3Aa in the process of immune priming, we used a strain that was obtained by conjugation from the naturally non-priming *Bt407* strain but that carries the plasmid encoding for Cry3Aa (*B. thuringiensis 407gfp-neocry+* [18] here called *Bt407+*), and a strain of *Btt* that was cured of that plasmid (*Btt-*). *Btt* supernatants induced a significant effect on survival. However, supernatants of *Btt-* failed to induce priming in *T. castaneum* larvae, whereas larvae primed with supernatants of *Bt407 +* showed a trend for an increased survival, always compared to fresh *Bt* medium (figure 2*a*, electronic supplementary material, figure S5 and file S2).

This indicates that Cry3Aa might be important for oral immune priming. Greenwood *et al.* [15] found some genes, potentially associated with the Cry toxin, upregulated in primed *T. castaneum* larvae, including the hexamerin gene. In other insects, this gene family helps the host resist Cry toxins either directly by interacting with the toxin or indirectly by inducing cell proliferation [34,35]. Supernatant proteins that remain intact after the production of ‘flour disks’ for the oral priming treatment (mixing with flour, drying at 30°C for 24 h) are likely candidates. Cry toxins are known to be environmentally stable, which is also relevant for their use in commercial insecticides. Although Cry toxin crystals would not pass the filters used during preparation of the priming diet, monomers that pass the filters might be sufficient to elicit a priming response. Such monomers could accumulate early in the cultures, as the expression of Cry3Aa is sporulation-independent [36,37]. Indeed, priming was evident in supernatants collected as early as 12 h after culture start compared to medium control (figure 2*b*, electronic supplementary material, figure S6).

It has to be noted that the Cry-carrying plasmid in the otherwise non-priming inducing bacterium (*Bt407+*) does not fully restore priming, suggesting that additional compounds are involved. One example in the *Btt* supernatant could be spore-coat-associated proteins, which may be recognized by the host as *Btt*-specific (figure 1*b*). Chitinase D, an enzyme that hydrolyses chitin in the *T. castaneum* peritrophic matrix, was also differentially expressed. Chitinases may contribute to B*. thuringiensis* toxicity [38,39], potentially synergizing with Cry3Aa [40,41]. Furthermore, we cannot rule out that molecules other than proteins, like metabolites, or bacterial cell wall components such as peptidoglycans, are involved in immune priming induction.

Also, proteins in the *Bt407-* supernatant might suppress a priming response. Proteins like immune inhibitor A and neutral protease B are more abundant in *Bt407-* supernatant compared to *Btt* supernatant (figure 1*b*). These proteins have been described as immunomodulatory in other organisms [42–44]. However, we argue that a specific immune response against pathogen-associated proteins, leading to immune priming, is more likely. Our data support this as we showed that without the Cry3Aa-bearing plasmid, *Btt* supernatants failed to induce immune priming (figure 2*a*).

Our results identify the Cry toxin as a strong candidate for triggering the described oral priming response in our model system. In other organisms, sublethal doses of Cry toxins have been shown to provoke host responses like increased midgut epithelium renewal, vesicle trafficking, autophagy and apoptosis [39,45–47]. Such damage reactions could release ‘danger’ signals and thereby trigger an immune reaction [48,49]. An interesting example in this context is the oral immune priming observed in mosquitoes [50], where disruption of midgut barriers by *Plasmodium* ookinetes enables the resident microbiota to reach the haemocoel, which in turn provokes a differentiation of haemocytes, making the host more resistant against subsequent *Plasmodium* infections. However, it should be noted that the specificity observed in our system may need additional explanations. Futo *et al*. [14] showed that two virulent strains of *B. thuringiensis* encoding for two different Cry toxins induced a priming response in *T. castaneum* in a strain-specific manner and failed to prime *T. castaneum* larvae against the other strain. Such specificity might be caused by differential host reactions to different Cry toxins, or via a combination of a damage response with additional specificity-conferring mechanisms. Further work is needed to test for possible connections between different Cry toxins and specific immune priming. Our study provides a basis for future work on how the insect immune system can memorize and respond specifically to certain pathogens.

## Data Availability

Mass spectrometry data are available via the JPOST repository: https://repository.jpostdb.org/preview/11322683365361efa5e7b7, access key 2082 [51]. Additional methods, results, data and analysis code are available in the electronic supplementary material [52].
